# More is less: Glucocorticoid-sparing agents in polymyalgia rheumatica

**DOI:** 10.1177/1759720X261480000

**Published:** 2026-09-15

**Authors:** Andriko Palmowski, Frank Buttgereit, Anja Strangfeld, Zhivana Boyadzhieva

**Affiliations:** 1 Department of Rheumatology and Clinical Immunology, Charité – Universitätsmedizin Berlin, Berlin, Germany; 2 Section for Biostatistics and Evidence-Based Research, the Parker Institute, Bispebjerg and Frederiksberg Hospital, University of Copenhagen, Copenhagen, Denmark; 3 Epidemiology and Health Services Research, German Rheumatism Research Center (DRFZ), A Leibniz Institute, Berlin, Germany; 4 Berlin Institute of Health (BIH) at Charité – Universitätsmedizin Berlin, Berlin, Germany; 5 Translational Rheumatology, German Rheumatism Research Center (DRFZ), A Leibniz Institute, Berlin, Germany

**Keywords:** polymyalgia rheumatica, glucocorticoids, toxicity, adverse events, DMARD

## Abstract

Polymyalgia rheumatica (PMR) is one of the most common inflammatory rheumatic diseases in older adults. Glucocorticoids (GC) are very effective in treating PMR, but their use is associated with adverse events such as infections, osteoporosis or ophthalmological diseases. In patients with relapsing PMR and long-term GC dependency or those with an initial high risk of GC-related toxicity, GC-sparing agents can be used to minimize GC exposure. Several substances have been tested in clinical trials. Methotrexate was commonly used in the past decades, but the interleukin (IL)-6 receptor inhibitor sarilumab is the first (and currently only) GC-sparing agent approved for use in PMR in the United States and Europe. Sarilumab was able to decrease cumulative GC exposure and increase the probability of achieving remission in a recent trial. In the foreseeable future, janus kinase inhibitors and IL-17 inhibitors might join the treatment armamentarium of GC-sparing agents in PMR. Additionally, the 11β-hydroxysteroid dehydrogenase type 1 inhibitor clofutriben has shown promise in protecting against GC toxicity. This review summarizes real-world evidence on GC use and associated toxicity in patients with PMR, puts data from relevant trials into context and explores future directions in the treatment of patients with PMR.

## Introduction

Polymyalgia rheumatica (PMR) is among the most common inflammatory rheumatic diseases in older adults. In the United Kingdom, about 0.85 % of people aged > 40 years are affected.^1^ Similar numbers are coming from the United States,^2^ while the disease seems to be much less common in people not from northern European descent. In South Korea, for example, the prevalence was estimated to be only around 0.008 %.^3^ The mean age at incidence is around 70 years,^4^ and people younger than 50 years are almost never affected. PMR affects women more commonly than men with a ratio of about 1:2-3.^1,2^

Bilateral pain and stiffness in the shoulders and hips, aggravated during nighttime and in the morning, are typical symptoms of PMR. The disease usually progresses quickly over a few days, and sometimes patients even report that the pain occurred suddenly from one day to the next. Pronounced morning stiffness is present in most patients with PMR. Patients may have accompanying unspecific symptoms such as fatigue, a feeling of illness, unintended weight loss, depression or low-grade fever,^5^ although high-grade fever should prompt the clinician to rule out other differential diagnoses and concomitant giant cell arteritis (GCA). GCA sometimes accompanies PMR, and some experts consider both diseases to belong to one overarching disease spectrum^6^: About 22% of patients with PMR have GCA at the time of diagnosis.^7^ A systematic review and meta-analysis of studies using positron emission tomography – computed tomography (PET-CT) imaging found an even higher prevalence of subclinical GCA in patients with PMR who had no cranial or ischemic symptoms (29 %).^8^ About half of patients with GCA, on the other hand, also suffer from PMR.^9^ Some patients who initially present with PMR may develop GCA during their disease course.

For decades, glucocorticoids (GC) have been the mainstay of treatment for PMR. This review highlights recent developments regarding GC-sparing agents in PMR and aims to explain why they are needed.

This review is based on a structured, non-systematic literature search. MEDLINE (via PubMed) was searched to identify relevant publications on PMR, with a focus on recent clinical trials, key observational studies, and reviews. Search terms included combinations of “polymyalgia rheumatica,” “PMR,” “treatment,” and “clinical trials.” Emphasis was placed on recent literature to reflect current developments in the field. In addition, this review incorporates evidence identified during prior comprehensive literature screening and data extraction conducted as part of the development of PMR management guidelines by German, Austrian, and Swiss rheumatology societies.^10^ Relevant studies from both sources were selected based on their clinical relevance and contribution to the topic.

## Why are glucocorticoid-sparing agents in patients with polymyalgia rheumatica needed?

GC have been used for decades to treat PMR. “Myalgic Syndrome with Constitutional Effects” was the landmark publication from 1957 in which Stuart Barber from Manchester coined the term ‘PMR’, and here he already described that patients with PMR had an immediate response to GC.^11^ Nowadays, recommendations from both Europe (European Alliance of Associations for Rheumatology) and the United States (American College of Rheumatology) suggest that GC be used in all patients with PMR at a starting dose between 12.5 and 25 mg/day prednisone equivalent,^12^ followed by a taper. A quick improvement in the signs and symptoms of PMR is typical after the initiation of GC, and a lack thereof is suggestive of alternative diagnoses. But although GC are effective in PMR, there is a need for alternative treatments because long-term GC use is relatively common and associated with relevant adverse events (AE).

While the immediate response to GC is beyond doubt, many patients experience relapses during GC tapering and require longer-term GC treatment. In a systematic review and meta-analysis of observational studies, 43% (95% confidence interval: 29-56%) of patients with PMR had at least one relapse during one year after diagnosis.^13^ This leads to prolonged GC use: In the same meta-analysis, after one, two and five years, 77%, 51% and 25% of observed patients were estimated to still be on GC, respectively.^13^ Figure 1 shows the estimated proportion of patients with PMR using GC over time. Of note, in current guidelines such as the 2024 German, Austrian and Swiss guidelines,^14^ GC monotherapy is usually recommended for a maximum of one year.

**Figure 1.**
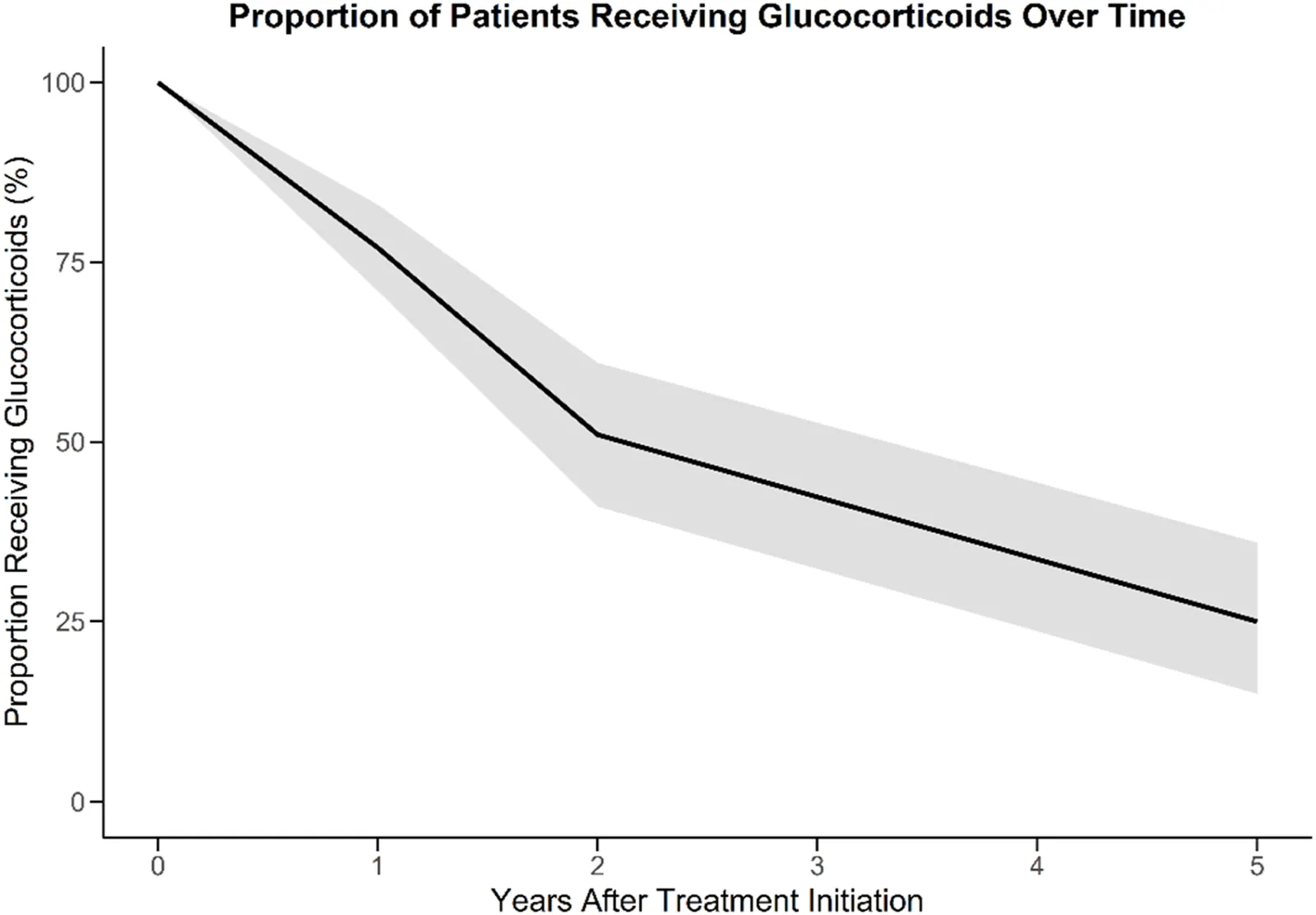
Own illustration based on data from the systematic review and meta-analysis of observational studies by Floris et al.^13^ The figure shows the proportion of patients with polymyalgia rheumatica receiving glucocorticoids over time in various real-world studies under different tapering regimens over five years from treatment initiation. The grey shadow shows the 95% confidence interval. Linear trends between time points for both point estimates and confidence intervals were assumed.

Prolonged GC use, can lead to a variety of AE,^15^ including arterial hypertension, diabetes, increase in body weight, depression, parchment-like skin, impaired wound healing, glaucoma and cataract, infections, myopathy, osteoporosis, and peptic ulcer disease, although some of these only occur during long-term or high dose treatment. Patients with PMR are particularly vulnerable to GC-related AE as they are typically of older age, which in turn is associated with comorbidities such as hypertension, diabetes or osteoporosis. For example, in a recent observational study in patients with PMR from Japan, 44%, 36%, 35% and 32% had hypertension, peptic ulcer disease, diabetes or osteoporosis at baseline, respectively.^16^ In a German cross-sectional analysis of patients with PMR with variable disease duration, 49% had hypertension, 17% had osteoporosis, and 17% had diabetes.^17^ All of these comorbidities can be caused or aggravated by GC.

Much evidence regarding GC-related AE in rheumatology stems from studies in patients with rheumatoid arthritis (RA). In a systematic review and meta-analysis of randomized trials with a duration of at least two years, the risk of infections was increased by 40% in patients with RA taking low dose GC (i.e., ≤7.5 mg/d prednisone equivalent) compared to placebo.^18^ In a subsequent individual participant data meta-analysis, about one additional kg of body weight gain was observed with low dose GC over two years while blood pressure was not affected.^19^ Those treated with low dose GC had a small decrease in bone mineral density at the lumbar spine but not at the femur (manuscript accepted for publication, RMD Open).^20^

In GCA (which is associated with PMR to a certain extent, as described above), a claims data analysis estimated 0.43 AE per patient-year of GC exposure,^21^ with cataract and bone disease (including osteoporosis and others) being the most common in this study (0.16 events per patient-year). The incidence of infections was similarly common in another study^22^ (mostly lower respiratory infections, conjunctivitis and herpes zoster). The absolute risk of incident diabetes mellitus was 13.7% in an analysis of electronic health records from the United States.^23^ Several studies have found that the prevalence of osteoporosis is much higher in patients with GCA compared to healthy controls.^24,25^ However, it is unclear whether evidence from patients with RA or GCA may be directly applied to patients with PMR, so studies on GC toxicity in patients with PMR are needed.

There is less direct evidence from studies in patients with PMR, and evidence from randomized trials is even rarer. Tanaka et al. recently performed a retrospective cohort study in Japan using electronic health records of 373 patients with PMR.^16^ Over one year, these patients received a median cumulative GC dosage of 2.5 g prednisone equivalent. Almost 50% had incident osteoporosis within one year after diagnosis, 30% had incident diabetes, and 15% had incident hypertension. All of these AE were more likely to occur in patients with higher GC doses. In another study from the United States,^26^ patients with PMR received a cumulative dose of 2.7 g of prednisone equivalents over one year, which is very similar to the Japanese data. Interestingly, in this study, the risks for diabetes, hypertension, hyperlipidemia and fractures were similar to age- and sex-matched controls; only the risk for incident glaucoma was increased by 70 %. A retrospective Italian study found that 39% of 58 patients with PMR, followed up over twelve months, had at least one GC-related AE. Comorbidity burden emerged as a main predictor: For every additional comorbidity, the risk of GC-related AEs increased by 27% (hazard ratio 1.27; 95% confidence interval 1.01 to 1.61; p = 0.044). Consequently, patients with more comorbidities at diagnosis should be monitored more closely for relevant AE and are candidates for earlier use of GC-sparing agents.

Whether the strongest driver for AE in patients with PMR is the disease itself or its treatment with GC has not yet been finally answered, and it is difficult to assess as virtually all patients with PMR receive GC. Systemic inflammation has been shown to increase the risk of fractures^27^ or cardiovascular events.^28,29^ Some studies associate higher cumulative GC doses and longer GC use with an increased risk, but it is likely that the results are in part confounded by indication, especially in studies that did not adjust for disease activity, severity, or systemic inflammation.^30^ The authors of this current article consider it likely that the answer lies in the middle, i.e., the risk increase in AE such as infections or osteoporosis in patients with PMR is the result of both the disease itself and GC toxicity.

In order to minimize GC exposure, various disease-modifying antirheumatic drugs (DMARDs) have been investigated in patients with PMR. According to current evidence, these GC-sparing agents can be considered in one of two scenarios. First, if patients with PMR relapse during GC taper, especially if more than one relapse occurs, as this is thought to increase cumulative GC exposure and subsequent GC toxicity. Second, GC-sparing agents can be used in new-onset disease if patients have a high risk for GC-induced AE, for example in patients who have a history of severe osteoporosis which is likely to exacerbate with GC.

## Conventional synthetic disease-modifying antirheumatic drugs in patients with polymyalgia rheumatica

Until recently, most data on GC-sparing effects was available for methotrexate (MTX). Earlier studies have noted its GC-sparing potential while also indicating similar or higher remission rates compared to GC monotherapy.^31–34^ In 2004, Caporali et al. reported a randomized trial which yielded lower median cumulative prednisone dose during treatment with MTX compared to placebo after 76 weeks (2.1 g vs 2.97 g).^31^ In a small Iranian trial, MTX increased the probability of remission at week 44 (risk ratio 5.22 [1.28 to 21.29]) and slightly reduced the cumulative GC dose (3.2g vs 3.7g), but without any difference in the relapse rate (34.8% vs 33.3%).^33^ A very small randomized trial in 21 patients, on the other hand, found no differences between MTX and placebo concerning time to and duration of remission, relapse rate or cumulative prednisone dose.^34^ Several retrospective cohort studies with a variable risk of bias showed no clear benefit of MTX on the median GC dose, flare incidence or remission rates.^35–37^ Interpretation of efficacy data is additionally complicated by the usage of relatively low weekly doses (≤10mg) in some of the earlier trials.^31–34^ Conference abstracts from a recent RCT on MTX and GC-free remission were incongruous^38,39^; the study still awaits publication in a peer-reviewed scientific journal.

To summarize, the available evidence on MTX in patients with PMR is conflicting, in part due to different outcome measures, populations and treatment strategies studied. MTX can still be considered a treatment option in PMR but will likely be used less commonly in the future since interleukin (IL)-6 inhibitors have been officially approved to treat PMR in the United States and Europe.

## Biologic and targeted synthetic disease-modifying antirheumatic drugs for polymyalgia rheumatica

While initial trials investigating tumor necrosis factor α inhibitors in patients with PMR were disappointing,^40,41^ growing evidence from trials and observational studies in other agents has introduced a treatment paradigm shift from csDMARDs to biologic therapies. Recent data on IL-6 inhibition have become a strong driving force, which is already reflected in the German, Austrian and Swiss^10^ as well as the French guidelines for PMR.^42^ New EULAR recommendations for PMR and large vessel vasculitis are currently in development (QoC023). Figure 2 shows a timeline of various trials investigating GC-sparing agents in PMR.

**Figure 2.**
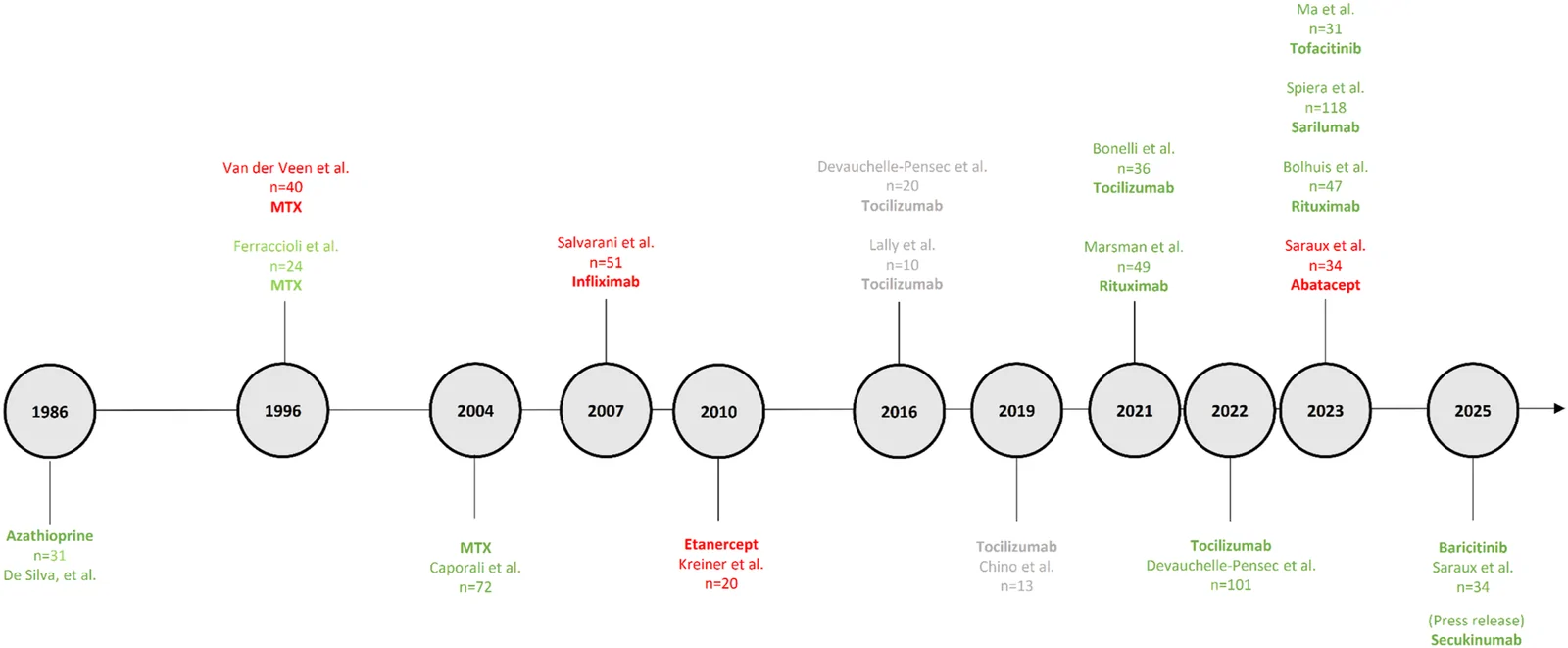
Important interventional studies in patients with polymyalgia rheumatica. Randomized trials which met their primary endpoint are colored green, those in red did not. Non-randomized interventional studies are highlighted in grey. MTX, methotrexate.

Sarilumab is the only GC-sparing agent approved for PMR in Europe and the United States.^43,44^ The FDA and EMA approved sarilumab after the SAPHYR trial in 118 patients with relapsing PMR showed significantly higher remission rates at 52 weeks compared to placebo (28.3% vs 10.3%) as well as a reduction in the median cumulative GC dose (777 mg vs 2,044 mg).^45^ However, these outcomes reflect on-treatment efficacy at 52 weeks, and data on durability after treatment discontinuation are limited. Post hoc analyses with short-term follow-up suggest that disease control may be maintained in a subset of patients after stopping sarilumab, but a substantial proportion require rescue therapy or have recurrent disease, and the true relapse rate after discontinuation remains uncertain.

During the trial, several instruments were used to assess the effect of sarilumab on health-related quality of life.^46^ It was shown that the drug led to significant improvements in the Short Health Form Survey (SF-36) Physical and Mental Component Summary (PCS 7.65 vs 2.87, p=0.02; MCS 3.04 vs -1.7, p=0.03). Patients in the sarilumab group also reported clinically important improvements in the SF-36 more frequently than those receiving placebo (odds ratio 3.46 [95% confidence interval 1.16–10.62], p=0.020).

Tocilizumab, another IL-6 inhibiting bDMARD, has also been studied in patients with PMR. The PMR-SPARE trial showed that tocilizumab was significantly superior to placebo regarding sustained GC-free remission at 16 weeks (63.2% vs 11.8%), time to relapse (130 vs 82 days) and cumulative GC dose (781 mg vs 1,290 mg) in newly diagnosed patients.^47^ Intravenous tocilizumab led to higher rates of disease remission at week 24 (67% vs 31%) and to a lower median daily GC dose (3.8 vs 6.1mg/day) in the SEMAPHORE trial, which enrolled patients with GC-dependent PMR.^48^ While promising, the available data covered only a relatively short time span as both RCTs offered a follow-up of merely 24 weeks. Recently, a longer-term follow-up study of the SEMAPHORE trial was published. It investigated the rates of sustained remission at week 48 after tocilizumab was withdrawn at week 24.^49^ Interestingly, only a minority of patients remained relapse-free after discontinuation (2 out of 26, i.e., 7.7%). The median time to relapse after tocilizumab withdrawal was 15 weeks (95% confidence interval 8.7-24). These results suggest effective remission induction but questionable sustainment thereof, raising the question of optimal treatment duration.

Another open question pertains to IL-6 inhibiting monotherapy (i.e., without GC). In the TENOR trial, all 20 PMR patients (both newly diagnosed and relapsing) achieved low disease activity after receiving monthly tocilizumab infusions without GC.^50^ In another study, remission was achieved only by a third of newly diagnosed patients (4 out of 13, 31%) at week 12, however, at week 52, most patients had achieved remission (9 out of 13, 69%).^51^ The treatment regimen here differed substantially – patients received intravenous tocilizumab fortnightly for 2 months and monthly for another 10 months. In both studies disease activity was measured according to the PMR activity score (PMR-AS): a composite score including CRP, patient´s pain assessment, physician´s global assessment, duration of morning stiffness as well as the ability of elevating the upper limbs.^52^

Although only sarilumab is approved in the U.S. and Europe, it seems reasonable to consider both IL-6 receptor inhibitors similarly effective. No evidence suggests the need for special caution regarding efficacy or safety favoring one drug over the other.^10^ IL-6 inhibitions seems to be a relatively safe treatment, although longer-term data from patients with PMR are still lacking.

In the SAPHYR trial 15% vs 0% of the patients in the sarilumab and placebo groups respectively developed neutropenia (p<0.05), however without associated infections. Arthralgia, diarrhea and insomnia were reported in >10% in the sarilumab group, however without statistical significance compared to placebo. In the tocilizumab trials neutropenia was also reported, but rates between groups did not differ significantly. There were no cases of gastrointestinal perforation, a recognized risk with IL-6 inhibitors.

Apart from IL-6 inhibitors, several other biologic agents have been explored in PMR. B-cell depleting therapy with rituximab was assessed in the BRIDGE-PMR trial^53^ and its one-year extension study.^54^ Both newly diagnosed and GC-dependent patients received a single infusion of 1000 mg rituximab. The GC-free remission rates were higher in the intervention group both at 21 and at 52 weeks (48% vs 21%, p=0.049 and 52.1% vs 20.8%, p=0.04, respectively) and the cumulative GC dose was lower at 52 weeks (1595mg vs 2302 mg, p=0.044). There were two cases of infection in the rituximab group (diverticulitis and herpes zoster) versus six in the placebo group, none of which led to treatment discontinuation. Currently, two larger RCTs with longer follow up are ongoing for both recently diagnosed (REDUCE-PMR-1) and relapsing patients (REDUCE-PMR-2).

T-cell activation also plays an important role in the pathophysiology of PMR,^55^ which motivated studies of abatacept, an inhibitor of CD28-mediated T-cell co-stimulation. The ALORS trial was a proof-of-concept randomized double-blind study of abatacept in patients with newly diagnosed active PMR.^56^ In this trial, the rates of GC-free low disease activity at week 12 were not significantly different to placebo (50% vs 22%, p=0.15), although the trial may have been underpowered to detect such a difference as it narrowly missed statistical significance. The changes in the PMR-AS were also mostly driven by CRP reduction, none of the other score components improved significantly (for example, pain VAS 1.6 vs 2.1, p=0.9; morning stiffness 49.7 min vs 58.9 min, p=0.96). Rates of adverse events were higher in the placebo group (50 vs 83%), without reaching statistical significance and without signaling new safety concerns.

Janus kinase inhibitors (JAKi) have also been regarded as a potentially viable treatment option since IL-6 downward signaling usually follows the JAK/STAT pathway. Tofacitinib monotherapy versus GC in treatment-naïve PMR patients was investigated in a 24-week RCT (EAST-PMR trial).^57^ In this trial, all patients in both groups achieved remission at weeks 12 and 24. Tofacitinib alongside GC led to remission in 85.7% of treated patients at week 48 in an open-label phase 2 study (12 out of 14, p=0.014). In the BACHELOR study in treatment-naïve patients, baricitinib was more effective compared to placebo in achieving remission without oral GC rescue therapy at week 12 (77.8% vs 13.3%, p<0.001).^58^ Even after a dose reduction past week 12 (from 4mg to 2mg), most patients maintained treatment response (16 out of 18, 90%), and most participants remained in remission until the end of the observational period of 36 weeks, despite stopping baricitinib after 24 weeks. Additional signals on possible efficacy of JAKi in PMR stem from the recent SELECT-GCA trial in upadacitinib plus a 26-week GC taper which showed higher remission rates at 52 weeks compared to placebo (46.4% vs 29%, p=0.002)^59^ Similarly to other drug classes, potential AE must be taken into account when considering JAKi. This is especially important in patients with a history of malignancy or cardiovascular events since a large trial in patients with RA found an increased risk of such AE.^60^ In the EAST-PMR trial no severe adverse events occurred, rates of AEs were higher in the baricitinib group in the BACHELOR trial: infections 33 vs 6%, musculoskeletal and connective tissue disorders (such as arthralgia, back pain) 72 vs 25%, without reaching statistical significance. There were no deaths and no major adverse cardiovascular events in either study group.

The IL-17A inhibitor secukinumab is currently under investigation for its use in PMR. A phase III trial (REPLENISH; NCT05767034) has reportedly met the primary endpoint and all secondary endpoints according to a press release.^61^ Secukinumab has previously led to higher sustained remission rates in GCA patients compared to placebo at week 28 (70% vs. 20%) with effects lasting 52 weeks (59% vs. 8%). A post hoc analysis of the subgroup with PMR symptoms from the same study revealed more frequent sustained remission rates at week 52 for secukinumab vs placebo (58.3% vs 12%).^62,63^

Further ongoing trials enrolling patients with PMR are listed in Table 1.

**Table 1.** Current trials in patients with PMR.

Study ID	Name	Design	Population	Intervention	Control	Status
ISRCTN17828080	STERLING-PMR	Phase 3, parallel-group, open-label RCT	Relapsing PMR	MTX + GC LEF + GC, if MTX not tolerated	GC	Recruting
NCT03576794	PMRLEFRCT	Phase 3, double-blind RCT	Newly diagnosed PMR	Leflunomide + GC	Placebo + GC	Recruiting
NCT06172361	ITTGPMR	Phase 3, open-label RCT	Newly diagnosed PMR	Tofacitinib + GC	GC	Recruiting
NCT06331312	REPLENISH-EXT	Phase 3, open-label, single-group assignment	Relapsing PMR	Secukinumab	none	Recruiting
NCT05767034	REPLENISH	Phase 3, double-blind RCT	Relapsing PMR	Secukinumab + GC	Placebo + GC	Completed Feb 2026
NCT05533125	REDUCE-PMR-1	Phase 3, double-blind RCT	Newly diagnosed PMR	Rituximab	Placebo	Completed Feb 2026
NCT05533164	REDUCE-PMR-2	Phase 3, double-blind RCT	GC-dependent PMR	Rituximab	Placebo	Active, not recruiting
NCT05636501	-	Open-label RCT	Newly diagnosed PMR	Treat-to-target GC Taper (nurse led)	GC taper following usual care (GP-led)	Active, not recruiting

PMR, polymyalgia rheumatica; RCT, randomized controlled trial; GC, glucocorticoid; GP, general practitioner.

## Future perspectives

The data presented above create new horizons in the treatment of PMR and open up the possibility of steering away from GC in the future by using biologic or tsDMARDs. However, many questions remain unanswered, for example, whether it would be cost-effective to co-treat all patients with new-onset PMR with a GC-sparing agent. Annual treatment costs of sarilumab and tocilizumab are substantially higher than those of conventional oral GC — by several orders of magnitude depending on the healthcare system and applicable pricing. The cost-effectiveness of these agents is likely to depend heavily on patient selection: in patients with frequently relapsing or GC-dependent disease, the reduction in cumulative GC exposure and associated AE (hospitalizations, fracture management, diabetes care) may partially offset drug acquisition costs. Conversely, routine use in all new-onset PMR patients - the majority of whom achieve remission on GC alone - seems unlikely to be cost-effective without further stratification by risk of GC toxicity or relapse. Future research should include prospective pharmacoeconomic studies and long-term budget impact analyses, ideally conducted from both payer and societal perspectives, to inform guideline recommendations and reimbursement decisions. The optimal treatment duration of GC-sparing agents is currently unknown as well and might impact cost-effectiveness.

To make things even more complicated, 11β-Hydroxysteroid dehydrogenase type 1 inhibitors (HSD-1i) have recently been investigated in clinical trials^64^ and might favorably alter the benefit-risk ratio of GC in the future. HSD-1 regulates intracellular GC activity by converting inactive GC into active isoforms. Inhibition of HSD-1 might reduce GC-related toxicity at target organs such as the bone while preserving therapeutic efficacy. Coadministration of clofutriben, an HSD-1i, improved markers of GC toxicity in a recent RCT,^64^ e.g., osteocalcin, triglycerides and cholesterol.

In any case, long-term follow-up studies of these new agents in larger PMR populations are dearly needed to add important safety data. A registry of patients with PMR, especially of those who require GC-sparing agents, could provide important answers.

## Limitations

This is a review of recent evidence on the pharmacologic treatment of PMR with GC and GC-sparing agents, including a discussion on both safety and efficacy. As described above, no systematic literature review was performed; instead, this is a narrative review, and relevant studies might have been missed by our literature search.

## Conclusion

PMR can lead to prolonged GC use, which is associated with a variety of AE. Patients with relapsing disease and those with new-onset disease who have a high risk for GC toxicity should be considered for co-treatment with GC-sparing treatments. Several agents have been tested in clinical trials. Currently, the IL-6 receptor inhibitor sarilumab is the only substance approved for PMR in the United States and Europe. Sarilumab was able to decrease cumulative GC exposure and increase the probability of achieving remission in a recent trial. In the foreseeable future, other agents such as JAKi, IL-17 inhibitors or HSD-1i might join and expand treatment options in PMR.
